# Real‐world safety and efficacy data of ipilimumab in Japanese radically unresectable malignant melanoma patients: A postmarketing surveillance

**DOI:** 10.1111/1346-8138.15388

**Published:** 2020-06-08

**Authors:** Naoya Yamazaki, Yoshio Kiyohara, Hisashi Uhara, Tetsuya Tsuchida, Keiko Maruyama, Naoki Shakunaga, Eijun Itakura, Akira Komoto

**Affiliations:** ^1^ Department of Dermatologic Oncology National Cancer Center Hospital Tokyo Japan; ^2^ Dermatology Division Shizuoka Cancer Center Hospital Nagaizumi Japan; ^3^ Department of Dermatology Sapporo Medical University Sapporo Japan; ^4^ Department of Dermatology Saitama Medical University Moroyama Japan; ^5^ Bristol‐Myers Squibb K.K. Tokyo Japan

**Keywords:** cytotoxic T‐lymphocyte‐associated antigen 4, efficacy, ipilimumab, malignant melanoma, safety

## Abstract

Treatment with immune checkpoint inhibitors has improved prognosis among patients with cutaneous melanoma, but there are still unmet medical needs in Japan, especially for mucosal melanoma and acral lentiginous melanoma (ALM) subtypes. Ipilimumab, a fully human monoclonal antibody that specifically blocks cytotoxic T‐lymphocyte‐associated antigen 4 and potentiates antitumor T‐cell response, was approved in Japan in 2015 for the treatment of radically unresectable malignant melanoma. This postmarketing surveillance (prospective, non‐interventional, multicenter, observational study) evaluated the safety (occurrence of adverse drug reactions [ADR]) and efficacy (overall survival [OS]) of ipilimumab in a real‐world setting in Japan. All patients with radically unresectable malignant melanoma undergoing treatment with ipilimumab in Japan during the registration period between August 2015 and February 2017 were enrolled. In total, 547 patients were analyzed; 67.5% were 60 years old or more, 85.7% had an Eastern Cooperative Oncology Group performance status of 0–1, 50.3% had melanoma of the skin (mainly of the ALM subtype) and 73.5% had negative *BRAF* mutation status. Most patients had experienced recurrence and received multiple treatments. The overall incidence of ADR and serious ADR was 69.5% and 40.8%, respectively. The most common ADR and serious ADR were liver disorder, colitis and diarrhea. The most common ADR of special interest were liver‐related ADR (22.5%), skin‐related ADR (22.1%), gastrointestinal‐related ADR (20.3%) and endocrine system‐related ADR (16.3%). Most of these events had recovered or were in remission by the last evaluation. The median OS was 7.52 months (95% confidence interval, 6.47–8.74). Median OS was 6.31 and 8.44 months in patients with mucosal melanoma and melanoma of the skin; 9.43 and 3.75 months in patients with and without ADR; and 10.32 and 6.11 months in patients with and without serious ADR, respectively. Ipilimumab was tolerable and showed efficacy in improving OS for these patients.

## Introduction

While malignant melanoma is a common skin cancer among Caucasians, it is considered a rare type of skin cancer for non‐Caucasians.^1^ The incidence of malignant melanoma in Japan in 2011–2013 was 1.75/100 000 persons per year.^2^ The number of Japanese patients with skin cancer reportedly increases rapidly between the ages of 40 and 49 years and peaks at the age of 60 years.^3^ Consequently, with the continuous growth of the aging population in Japan, the mortality rate associated with skin cancer is also increasing.^2^ Notably, approximately 40% of the deaths caused by skin cancer in Japan are attributable to malignant melanoma.^4^

There are remarkable differences between malignant melanoma in Japanese populations and malignant melanoma affecting Caucasians. Some major differences are the overall frequency of cases (more frequent in Caucasian than Japanese populations),^2^ proportions of tumor subtypes (higher frequency of acral lentiginous melanoma [ALM] and mucosal melanoma compared with Caucasians), types of mutations (lower proportions of *BRAF* mutations compared with Caucasians) and tumor site (sole of the foot in Japanese patients compared with the trunk in Caucasian patients).^4^, ^5^, ^6^

Prior to the introduction of immune checkpoint inhibitors, the prognosis of patients with melanoma was poor.^2^, ^3^ Although advances in treatment with immune checkpoint inhibitors have resulted in improved prognosis among patients with cutaneous melanoma, there are still unmet treatment needs in Japan, particularly for mucosal melanoma and ALM subtypes.^7^

Ipilimumab is a fully human monoclonal antibody of the immunoglobulin (Ig)G1 isotype that specifically binds to anti‐cytotoxic T‐lymphocyte‐associated antigen 4 (CTLA‐4) and augments the antitumor response.^8^ Improvements in overall survival (OS) were observed in the global phase III study among previously treated patients with metastatic melanoma treated with a total of four doses of ipilimumab 3 mg/kg, every 3 weeks.^9^ Thus, ipilimumab was approved for melanoma as monotherapy (3 mg/kg, every 3 weeks for four doses) by the US Food and Drug Administration and the European Medicines Agency in 2011. In Japan, ipilimumab was approved in 2015 for the treatment of radically unresectable melanoma patients based on the results of the global phase III study^9^ and a Japanese phase II study.^10^ In the Japanese phase II study,^10^ the best overall response rate (ORR) was 10% (95% confidence interval [CI], 1.2–31.7), median OS was 8.71 months (95% CI, 3.71–not reached) and median progression‐free survival (PFS) was 2.74 months (95% CI, 1.25–2.83). Twelve patients (60%) had at least one drug‐related adverse event (AE), and 12 patients (60%) reported immune‐related adverse events (irAE).

As there were limited data on the safety and efficacy of ipilimumab among Japanese patients with radically unresectable melanoma, the Japan Ministry of Health requested the marketing authorization holder (Bristol‐Myers Squibb, Tokyo, Japan) to conduct a postmarketing surveillance (PMS) to provide data on ipilimumab use for the approved indication in a real‐world setting. The primary objectives of this postmarketing surveillance were to evaluate safety in terms of the occurrence of adverse drug reactions (ADR) and ADR of special interest (ADRI), assess the efficacy of ipilimumab based on OS, and identify factors that may affect the safety and efficacy of ipilimumab for Japanese patients with radically unresectable malignant melanoma in a real‐world setting based on the conditions of its approval.

## Methods

### Study design, patients and treatment

This was a prospective, non‐interventional, non‐controlled, multicenter (146 institutions), observational study (all‐case postmarketing surveillance; ClinicalTrials.gov Identifier, NCT02717364). The registration period of all Japanese patients with radically unresectable malignant melanoma was from August 2015 to February 2017 and the survey implementation period was from August 2015 to January 2019.

The study was conducted in accordance with Japanese regulatory requirements stipulated in Good Post‐marketing Study Practice,^11^ and approval from an ethics committee and written informed consent from the patients were not mandated as per the ministerial ordinance. Patients who had received at least one dose of ipilimumab were enrolled in the study by their treating physician, and each patient was followed up for 12 months. All patients with radically unresectable malignant melanoma treated with ipilimumab during the registration period were included in this postmarketing surveillance. There were no prespecified exclusion criteria.

This was a non‐interventional study; thus, ipilimumab treatment was prescribed by the treating physicians under routine, daily practice, in compliance with the recommendations in the Japanese prescribing information.^12^ The approved ipilimumab dose was 3 mg/kg of bodyweight administrated i.v. every 3 weeks for a total of four doses as a monotherapy. Treating physicians made treatment‐related decisions such as initiation, duration and discontinuation of treatment. If the treatment was not completed with four doses of ipilimumab, the patient was considered to have discontinued treatment.

### Data collection

The data collected in the case report forms (CRF) included patient demographics, clinical characteristics such as Eastern Cooperative Oncology Group performance status (ECOG PS), onset and recurrence, date of diagnosis, primary site, subtype, disease stage at baseline, M category, location of metastases, *BRAF* mutation status, prior therapy, complications, medical history, history of allergies, previous history of treatment for primary disease, hospitalization status, health‐care data related to ipilimumab treatment (date of administration, dose administrated, reasons for discontinuation and concomitant medications), laboratory test results (if available) and other examinations or procedures that patients underwent during the observation period.

### Study assessments

#### Safety

The safety of ipilimumab was evaluated based on the occurrence of ADR (AE) during 12 months after the initiation of ipilimumab treatment. An AE is any untoward medical occurrence in a patient or clinical investigation subject administrated a pharmaceutical product that does not necessarily have to have a causal relationship with this treatment. A serious AE is any untoward medical occurrence that, at any dose, results in death, is life‐threatening, requires inpatient hospitalization or causes prolongation of existing hospitalization, results in persistent or significant disability/incapacity, is a congenital anomaly/birth defect or is an important medical event. Non‐serious AE data were collected and confirmed for 6 months from the first dose of ipilimumab while only serious AE data were collected for 12 months of ipilimumab administration.

For AE and ADR, Preferred Terms (PT) and System Organ Class (SOC) terminology from the Medical Dictionary for Regulatory Activities, Japanese version (MedDRA/J version 21.1) were used. The seriousness of AE and ADR was determined by treating physicians based on the evaluation as per ICH E2A and E2D guidelines. The ADRI were gastrointestinal‐related ADR (diarrhea, colitis and gastrointestinal perforation), liver‐related ADR, skin‐related ADR, endocrine system‐related ADR (hypophysitis, hypopituitarism, hypothyroidism and adrenal insufficiency), peripheral neuropathy, renal disorders, interstitial lung disease and infusion reactions.

#### Efficacy

Survival at 12 months after the start of ipilimumab was regarded as effective, and the overall survival was estimated using the Kaplan–Meier method.

### Statistical analysis

The planned survey sample size was 400 patients, based on an estimated number of patients who would receive ipilimumab within 2 years of product launch. The 400‐patient target was considered to allow for the detection, with a probability of 95% or higher, of at least one event of the rarest AE, which were AE of special interest.

For safety, patients who received at least one dose of ipilimumab were included in the analysis. For efficacy, patients who received at least one dose of ipilimumab for the approved indication were included in the analysis (off‐label use would be excluded).

All statistical analyses were conducted using SAS version 9.4 (SAS Institute, Cary, NC, USA). Descriptive statistics including frequency distributions, means and standard deviations were used to analyze the data. The OS of patients who received treatment with ipilimumab was estimated using the Kaplan–Meier method. The statistical analysis method used was the log‐rank test. The level of significance was 95% and tests were two‐sided.

## Results

### Patient disposition and characteristics

**Figure 1:**
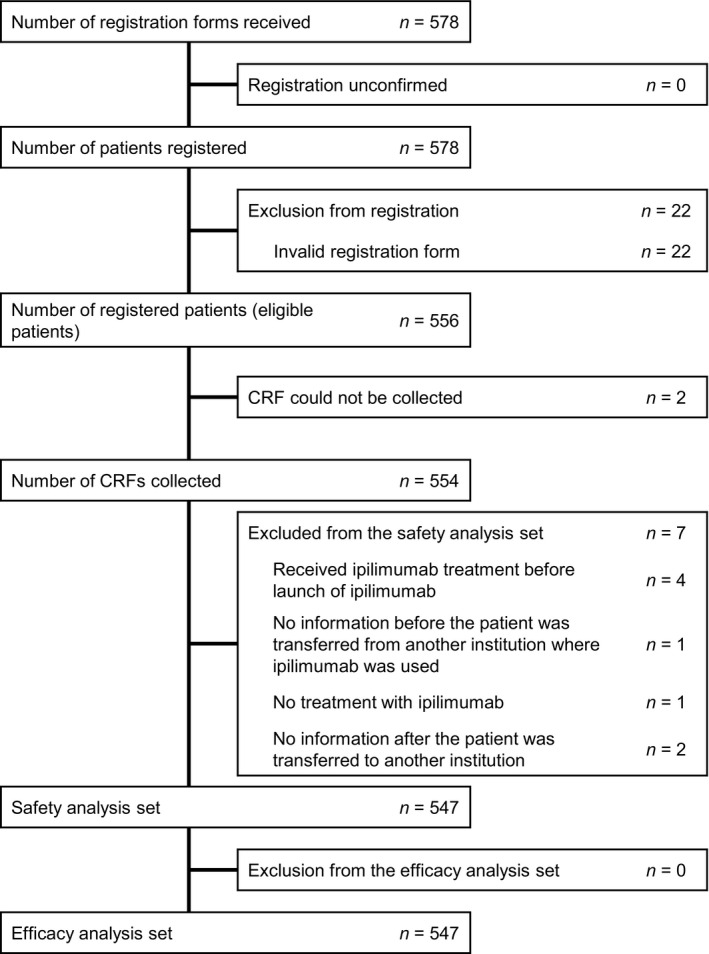
Patient disposition. CRF, case report form.

Of the 578 registered patients, 22 had invalid registration forms. Of the 556 eligible patients, CRF could not be collected for two patients; thus, 554 CRF were collected. Of these, seven patients were excluded from the safety and efficacy analysis sets; thus, 547 patients were analyzed for safety and efficacy (Fig. 1).

Table 1 summarizes the patient demographics and clinical characteristics at baseline. In the safety analysis set, approximately half of the patients were male (51.9%). Most patients were between 60 and 80 years of age (58.7%). The median (range) age was 66 (17–94) years. Most patients either had an ECOG PS of 0 (55.6%) or 1 (30.2%). The proportions of patients with ECOG PS 2, 3 and 4 were 7.9%, 4.4% and 1.8%, respectively. Nearly two‐thirds (64.7%) had experienced disease recurrence, and 50.3% had melanoma of the skin, followed by mucosal melanoma (32.9%) as the primary tumor site. Among those with melanoma of the skin, the most common tumor subtypes were ALM (19.6%), nodular melanoma (12.6%) and superficial spreading melanoma (7.5%).

**Table 1:** Patient backgrounds in the safety analysis set

	*n* = 547 *n* (%)
Sex	
Male	284 (51.9)
Female	263 (48.1)
Age category (years)	
<20	1 (0.2)
20 to <30	9 (1.6)
30 to <40	17 (3.1)
40 to <50	53 (9.7)
50 to <60	98 (17.9)
60 to <70	162 (29.6)
70 to <80	159 (29.1)
≥80	48 (8.8)
ECOG performance status category	
0	304 (55.6)
1	165 (30.2)
2	43 (7.9)
3	24 (4.4)
4	10 (1.8)
Unknown	1 (0.2)
First onset/recurrence	
First onset	190 (34.7)
Recurrence	354 (64.7)
Unknown	3 (0.5)
Not reported	0 (0.0)
Primary site category (at first onset)	
Melanoma of the skin	275 (50.3)
Mucosal melanoma	180 (32.9)
Ocular melanoma	33 (6.0)
Uveal melanoma	14 (2.6)
Others	33 (6.0)
Unknown	26 (4.8)
Not reported	0 (0.0)
Stage	
III	23 (4.2)
IV	251 (45.9)
Others	1 (0.2)
*BRAF* mutations	
No test conducted	73 (13.3)
Test conducted	474 (86.7)
Negative	402 (73.5)
Positive	69 (12.6)
Unknown	3 (0.5)
Subtype of melanoma of the skin	
Lentigo maligna melanoma	12 (2.2)
Superficial spreading melanoma	41 (7.5)
Nodular melanoma	69 (12.6)
Acral lentiginous melanoma	107 (19.6)
Others	18 (3.3)
Unknown/not reported	28 (5.1)
Complication (autoimmune disorders)	
No	474 (86.7)
Yes	73 (13.3)
Unknown/not reported	0 (0.0)
Previous history of treatment for primary disease	
No	17 (3.1)
Yes	530 (96.9)
Number of pharmacotherapies prior to ipilimumab	
No prior treatment (1st)	78 (14.3)
1 drug (2nd)	205 (37.5)
2 drugs and more (3rd and after)	264 (48.3)
Types of pharmacotherapy prior to ipilimumab	
No	78 (14.3)
Yes	469 (85.7)
Dacarbazine	250 (45.7)
Vemurafenib	46 (8.4)
Nivolumab	428 (78.2)
Others	88 (16.1)
Unknown/not reported	0 (0.0)
Surgery	
No	128 (23.4)
Yes	418 (76.4)
Unknown/not reported	1 (0.2)
Radiotherapy	
No	373 (68.2)
Yes	172 (31.4)
Unknown/not reported	2 (0.4)
Immunotherapy	
No	387 (70.7)
Yes	157 (28.7)
Unknown/not reported	3 (0.5)

ECOG, Eastern Cooperative Oncology Group.

The disease stage (melanoma of the skin only) was IV in 45.9% (251/547) of patients, the *BRAF* mutation status was negative in 73.5% (402/547) of patients and 96.9% of patients had a history of prior treatment. Almost half of the patients (48.3%) had received two or more previous treatments prior to ipilimumab (third‐line and later use of ipilimumab). Of those receiving drug therapy, 78.2% had received nivolumab, 45.7% had received dacarbazine and 8.4% had received vemurafenib.

### Dosing status

**Figure 2:**
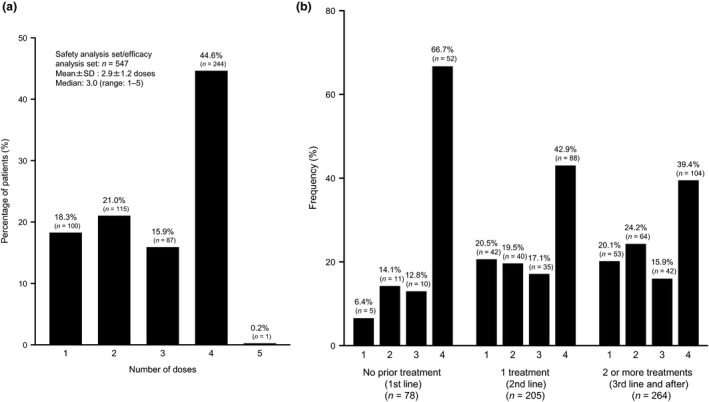
Dosing status of ipilimumab. (a) Total number of doses administrated. (b) Doses of ipilimumab by prior treatment. SD, standard deviation.

The mean (± standard deviation) number of total doses was 2.9 ± 1.2 and the median (range) was 3.0 doses (1–5) (Fig. 2a). The percentage of patients who completed four doses of ipilimumab was 44.6% (244/547 patients). One patient received five doses of ipilimumab. This patient experienced AE after the first dose and discontinued the administration of ipilimumab. Subsequently, the patient was transferred to another hospital and received four doses at that hospital, without presenting new ADR after the fifth dose. Figure 2(b) shows the number of ipilimumab doses received with respect to previous treatments (i.e. first‐, second‐, third‐line or further lines of treatment). Among patients with no prior treatment, 66.7% received four doses of ipilimumab, while among those with one and two or more previous treatments, 42.9% and 39.4% received four doses of ipilimumab, respectively.

The main reasons for treatment discontinuation by the number of ipilimumab doses received at the time of discontinuation are summarized in Table 2. With the first dose (*n* = 100), the most common reason for discontinuation was AE (40.0%), followed by disease progression (31.0%) and death due to primary disease (27.0%). Similarly, with the second (*n* = 115) and third doses (*n* = 87), the most common reasons for discontinuation were AE (53.0% and 66.7%, respectively) and disease progression (36.5% and 29.9%, respectively).

**Table 2:** Status of discontinuation of administration of ipilimumab

No. of doses administrated at time of discontinuation	Reasons for discontinuation, *n* (%)^†^						
	Adverse event	Disease progression or onset of new lesion	Death due to primary disease	No visit	Patient’s intention	Stable medical condition	Others
1 (*n* = 100)	40 (40.0)	31 (31.0)	27 (27.0)	3 (3.0)	3 (3.0)	1 (1.0)	2 (2.0)
2 (*n* = 115)	61 (53.0)	42 (36.5)	11 (9.6)	4 (3.5)	2 (1.7)	0 (0.0)	0 (0.0)
3 (*n* = 87)	58 (66.7)	26 (29.9)	3 (3.4)	2 (2.3)	1 (1.1)	0 (0.0)	1 (1.1)
Total (*n* = 302)^†^	159 (52.6)	99 (32.8)	41 (13.6)	9 (3.0)	6 (2.0)	1 (0.3)	3 (1.0)

^†^ When more than one reason for discontinuation of the administration of ipilimumab was provided, the data were tabulated for each reason for discontinuation.

### Safety

A total of 850 ADR were reported in 380 patients, with an incidence of 69.5% (380/547 patients) (Table 3). Common ADR were diarrhea in 12.4% (68/547 patients), liver disorder in 9.9% (54/547 patients) and colitis in 8.0% (44/547 patients). The incidence of serious ADR was 40.8% (223/547 patients). Common serious ADR were liver disorder in 6.9% (38/547 patients), colitis in 6.2% (34/547 patients) and diarrhea in 5.1% (28/547 patients).

**Table 3:** Occurrence status of adverse drug reactions with an overall frequency of more than 3% (safety analysis set, *n* = 547)

	Overall, *n* (%)	Serious, *n* (%)
No. of patients with adverse drug reactions (%)	380 (69.5)	223 (40.8)
No. of adverse drug reactions	850	365
Diarrhea	68 (12.4)	28 (5.1)
Liver disorder	54 (9.9)	38 (6.9)
Colitis	44 (8.0)	34 (6.2)
Pyrexia	42 (7.7)	16 (2.9)
Hypothyroidism	40 (7.3)	6 (1.1)
Rash	31 (5.7)	3 (0.5)
Hepatic function abnormal	30 (5.5)	22 (4.0)
Pruritus	24 (4.4)	1 (0.2)
Hypopituitarism	21 (3.8)	16 (2.9)
Adrenal insufficiency	20 (3.7)	16 (2.9)
Interstitial lung disease	19 (3.5)	13 (2.4)
Aspartate aminotransferase increased	19 (3.5)	7 (1.3)
Hypophysitis	19 (3.5)	16 (2.9)
Malaise	18 (3.3)	1 (0.2)
Alanine aminotransferase increased	18 (3.3)	8 (1.5)

Medical Dictionary for Regulatory Activities version 21.1.

Table 4 summarizes the incidence of ADRI. Common ADRI of special interest were liver‐related ADR (22.5%), skin‐related ADR (22.1%), gastrointestinal‐related ADR (20.3%) and endocrine system‐related ADR (16.3%). Of these, 13.3% of liver‐related ADR, 12.2% of gastrointestinal‐related ADR and 8.0% of endocrine system‐related ADR were serious.

**Table 4:** Occurrence status of adverse drug reactions of special interest

ADR of special interest	Safety analysis set, *n* = 547	
	No. of patients with ADR (%)	
	All	Serious
Liver‐related ADR	123 (22.5)	73 (13.3)
Skin‐related ADR	121 (22.1)	12 (2.2)
Gastrointestinal‐related ADR (diarrhea, colitis and gastrointestinal perforation)	111 (20.3)	67 (12.2)
Endocrine system‐related ADR (hypophysitis, hypopituitarism, hypothyroidism and adrenal insufficiency)	89 (16.3)	44 (8.0)
Interstitial lung disease	19 (3.5)	13 (2.4)
Peripheral neuropathy	11 (2.0)	6 (1.1)
Renal disorders	11 (2.0)	7 (1.3)
Infusion reaction	3 (0.5)	0 (0.0)

If a patient reported the same event more than once, it was counted only once. ADR, adverse drug reaction.

**Figure 3:**
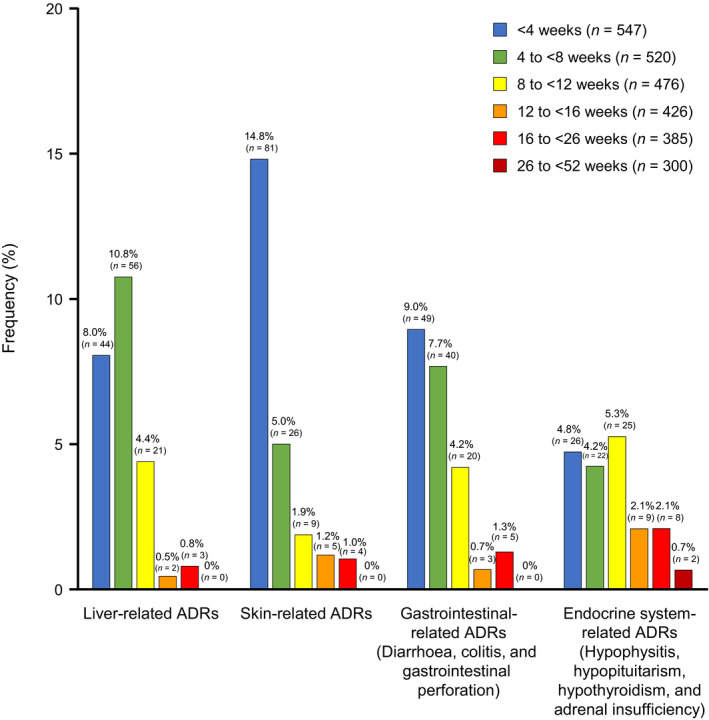
Timing of the first onset of adverse drug reactions (ADR) of special interest. Data from 26 to <52 weeks include only frequency of serious adverse drug reactions (ADRs).

Figure 3 shows the timing of the first onset of ADRI. In most cases, the onset of ADR occurred at the beginning of treatment at less than 8 weeks, especially for liver‐related, skin‐related and gastrointestinal‐related ADR. However, a different pattern of onset of ADR was shown for endocrine system‐related ADR, which tended to occur at 8 weeks or later.

**Figure 4:**
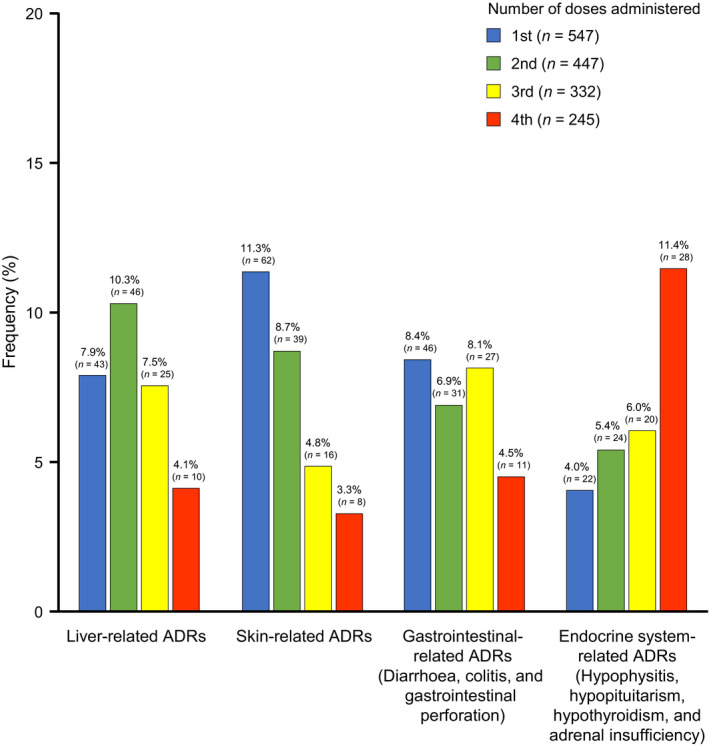
Incidence of adverse drug reaction at each dose. Each priority survey item has a duplicate count. When the same event occurred multiple times in the same case, it was counted using the first event. ADR, adverse drug reaction.

Figure 4 shows the incidence of ADRI at each dose. Liver‐related ADR tended to occur more frequently with the second dose, while skin‐related and, gastrointestinal‐related ADR tended to occur more frequently with the first dose. The frequency of endocrine system‐related ADR increased with an increasing number of doses, and the highest frequency was observed with the fourth dose.

A total of 152 liver‐related ADR were reported in 123 patients (Table 5a). The most common liver‐related ADR were liver disorder (*n* = 54, 9.9%), hepatic function abnormal (*n* = 30, 5.5%), aspartate aminotransferase (AST) increased (*n* = 19, 3.5%), and alanine aminotransferase (ALT) increased (*n* = 18, 3.3%). Of these, 6.9%, 4.0%, 1.3%, and 1.5% were serious, respectively. A total of 137 skin‐related ADR were reported in 121 patients (Table 6a). The most common skin‐related ADR were rash (*n* = 31, 5.7%), pruritus (*n* = 24, 4.4%) and skin disorder (*n* = 14, 2.6%). Of these, 0.5%, 0.2% and none were serious, respectively. A total of 129 gastrointestinal‐related ADR were reported in 111 patients (Table 7a). The most common gastrointestinal‐related ADR were diarrhea (*n* = 68, 12.4%), colitis (*n* = 44, 8.0%) and enterocolitis (*n* = 13, 2.4%). Of these, 5.1%, 6.2% and 2.0% were serious, respectively. A total of 124 endocrine system‐related ADR were reported in 89 patients (Table 8a). The most common endocrine system‐related ADR were hypothyroidism (*n* = 40, 7.3%), hypopituitarism (*n* = 21, 3.8%), adrenal insufficiency (*n* = 20, 3.7%) and hypophysitis (*n* = 19, 3.5%). Of these, 1.1%, 2.9%, 2.9% and 2.9% were serious, respectively.

**Table 5:** Summary of the (a) occurrence status and (b) outcomes of liver‐related events (safety analysis set, *n* = 547)

(a) Occurrence status of liver‐related events		
	Any ADR	Serious ADR
No. of patients with ADR (%)	123 (22.5)	73 (13.3)
No. of ADR	152	86
Liver disorder	54 (9.9)	38 (6.9)
Hepatic function abnormal	30 (5.5)	22 (4.0)
AST increased	19 (3.5)	7 (1.3)
ALT increased	18 (3.3)	8 (1.5)
γ‐GT increased	8 (1.5)	2 (0.4)
Blood ALP increased	7 (1.3)	3 (0.5)
Drug‐induced liver injury	5 (0.9)	4 (0.7)
Hepatic enzyme increased	5 (0.9)	–
Blood lactate dehydrogenase increased	3 (0.5)	–
Other^†^	3 (0.5)	2 (0.4)

(b) Outcomes by liver‐related event					
	Recovered	Remission	Not recovered	Death due to this event	Unknown
No. of ADR	88	40	23	1	0
Liver disorder	25	20	8	1	–
Hepatic function abnormal	19	9	2	–	–
AST increased	15	2	2	–	–
ALT increased	14	2	2	–	–
γ‐GT increased	4	3	1	–	–
Blood ALP increased	3	2	2	–	–
Drug‐induced liver injury	3	2	–	–	–
Hepatic enzyme increased	2	–	3	–	–
Blood lactate dehydrogenase increased	–	–	3	–	–
Other^†^	3	–	–	–	–

^†^ Hepatitis, hepatobiliary disease, blood bilirubin increased (one for each). Multiple events in the same case were tabulated for each event. ADR, adverse drug reaction; ALP, alkaline phosphatase; ALT, alanine aminotransferase; AST, aspartate aminotransferase; γ‐GT, γ‐glutamyl transferase.

**Table 6:** Summary of the (a) occurrence status and (b) outcomes of skin‐related events (safety analysis set, *n* = 547)

(a) Occurrence status of skin‐related events		
	Any ADR	Serious ADR
No. of patients with ADR (%)	121 (22.1)	12 (2.2)
No. of ADR	137	12
Rash	31 (5.7)	3 (0.5)
Pruritus	24 (4.4)	1 (0.2)
Skin disorder	14 (2.6)	–
Urticaria	10 (1.8)	–
Drug eruption	9 (1.6)	2 (0.4)
Erythema	9 (1.6)	1 (0.2)
Erythema multiforme	6 (1.1)	2 (0.4)
Leukoderma	5 (0.9)	–
Rash maculopapular	4 (0.7)	–
Palmar–plantar erythrodysesthesia syndrome	3 (0.5)	–
Dermatitis acneiform	2 (0.4)	–
Rash erythematous	2 (0.4)	–
Stevens–Johnson syndrome	2 (0.4)	2 (0.4)
Eczema	2 (0.4)	–
Dermatitis	2 (0.4)	–
Toxic skin eruption	2 (0.4)	–
Other^†^	10 (1.8)	1 (0.2)

(b) Outcomes by skin‐related event					
	Recovered	Remission	Not recovered	Death due to this event	Unknown
No. of ADR	76	42	15	0	4
Rash	17	10	2	–	2
Pruritus	13	8	1	–	2
Skin disorder	10	4	–	–	–
Urticaria	6	4	–	–	–
Drug eruption	5	4	–	–	–
Erythema	5	4	–	–	–
Erythema multiforme	6	–	–	–	–
Leukoderma	–	–	5	–	–
Rash maculopapular	2	1	1	–	–
Palmar–plantar erythrodysesthesia syndrome	2	1	–	–	–
Dermatitis acneiform	1	1	–	–	–
Rash erythematous	1	–	1	–	–
Stevens–Johnson syndrome	2	–	–	–	–
Eczema	2	–	–	–	–
Dermatitis	1	1	–	–	–
Toxic skin eruption	1	1	–	–	–
Other^†^	2	3	5	–	–

^†^ Alopecia areata, dermatitis exfoliative generalized, erythema nodosum, papule, pemphigoid, prurigo, rash macular, skin discoloration, achromotrichia acquired, papuloerythroderma of Ofuji (one for each). Multiple events in the same case were tabulated for each event. ADR, adverse drug reaction.

**Table 7:** Summary of the (a) occurrence status and (b) outcomes of gastrointestinal‐related events (safety analysis set, *n* = 547)

(a) Occurrence status of gastrointestinal‐related events		
	Any ADR	Serious ADR
No. of patients with ADR (%)	111 (20.3)	67 (12.2)
No. of ADR	129	77
Diarrhea	68 (12.4)	28 (5.1)
Colitis	44 (8.0)	34 (6.2)
Enterocolitis	13 (2.4)	11 (2.0)
Enteritis	2 (0.4)	2 (0.4)
Gastrointestinal perforation	1 (0.2)	1 (0.2)
Intestinal perforation	1 (0.2)	1 (0.2)

(b) Outcomes by gastrointestinal‐related event						
	Recovered	Recovered with sequelae	Remission	Not recovered	Death due to this event	Unknown
No. of ADR	82	1	40	3	1	2
Diarrhea	49	–	16	2	–	1
Colitis	24	–	18	1	–	1
Enterocolitis	8	–	5	–	–	–
Enteritis	1	–	1	–	–	–
Gastrointestinal perforation	–	1	–	–	–	–
Intestinal perforation	–	–	–	–	1	–

Multiple events in the same case were tabulated for each event. ADR, adverse drug reaction.

**Table 8:** Summary of the (a) occurrence status and (b) outcomes of endocrine system‐related events (safety analysis set, *n* = 547)

(a) Occurrence status of endocrine system‐related events		
	Any ADR	Serious ADR
No. of patients with ADR (%)	89 (16.3)	44 (8.0)
No. of ADR	124	63
Hypothyroidism	40 (7.3)	6 (1.1)
Hypopituitarism	21 (3.8)	16 (2.9)
Adrenal insufficiency	20 (3.7)	16 (2.9)
Hypophysitis	19 (3.5)	16 (2.9)
Secondary adrenocortical insufficiency	6 (1.1)	6 (1.1)
Thyroiditis	4 (0.7)	–
Blood thyroid‐stimulating hormone decreased	4 (0.7)	1 (0.2)
Blood thyroid‐stimulating hormone increased	3 (0.5)	–
Thyroxine free decreased	3 (0.5)	–
Thyroxine free increased	2 (0.4)	–
Other^†^	2 (0.4)	2 (0.4)

(b) Outcomes by endocrine system‐related event						
	Recovered	Recovered with sequelae	Remission	Not recovered	Death due to this event	Unknown
No. of ADR	37	1	43	39	0	4
Hypothyroidism	17	–	9	12	–	2
Hypopituitarism	5	–	8	8	–	–
Adrenal insufficiency	3	–	8	9	–	–
Hypophysitis	3	1	9	6	–	–
Secondary adrenocortical insufficiency	–	–	5	1	–	–
Thyroiditis	1	–	2	–	–	1
Blood thyroid‐stimulating hormone decreased	4	–	–	–	–	–
Blood thyroid‐stimulating hormone increased	2	–	–	1	–	–
Thyroxine free decreased	–	–	1	1	–	1
Thyroxine free increased	1	–	–	1	–	–
Other^†^	1	–	1	–	–	–

^†^ Adrenalitis, adrenocortical insufficiency acute (one for each). Multiple events in the same case were tabulated for each event. ADR, adverse drug reaction.

Of the 152 liver‐related ADR, 88 were recovered, 40 were in remission and 23 were not recovered at the time of assessment. One death due to liver disorder was reported (Table 5b). Of the 137 skin‐related ADR, 76 were recovered, 42 were in remission and 15 were not recovered at the time of assessment (of these, five cases were leukoderma and two with rash). No deaths related to skin‐related ADR occurred (Table 6b). Of the 129 gastrointestinal‐related ADR, 82 were recovered, 40 were in remission and three were not recovered at the time of assessment (two were diarrhea and one was colitis). One death due to intestinal perforation was reported (Table 7b). Of the 124 endocrine system‐related ADR, 37 were recovered, 43 were in remission and 39 were not recovered at the time of assessment (of these, 12, eight, nine and six were hypothyroidism, hypopituitarism, adrenal insufficiency and hypophysitis, respectively) (Table 8b).

### Efficacy

Among the 547 patients included in the efficacy analysis, treatment outcomes were death in 299 patients (54.7%), survival in 201 patients (36.7%) and outcomes unknown in 47 patients (8.6%) at 12 months after the start of ipilimumab.

**Figure 5:**
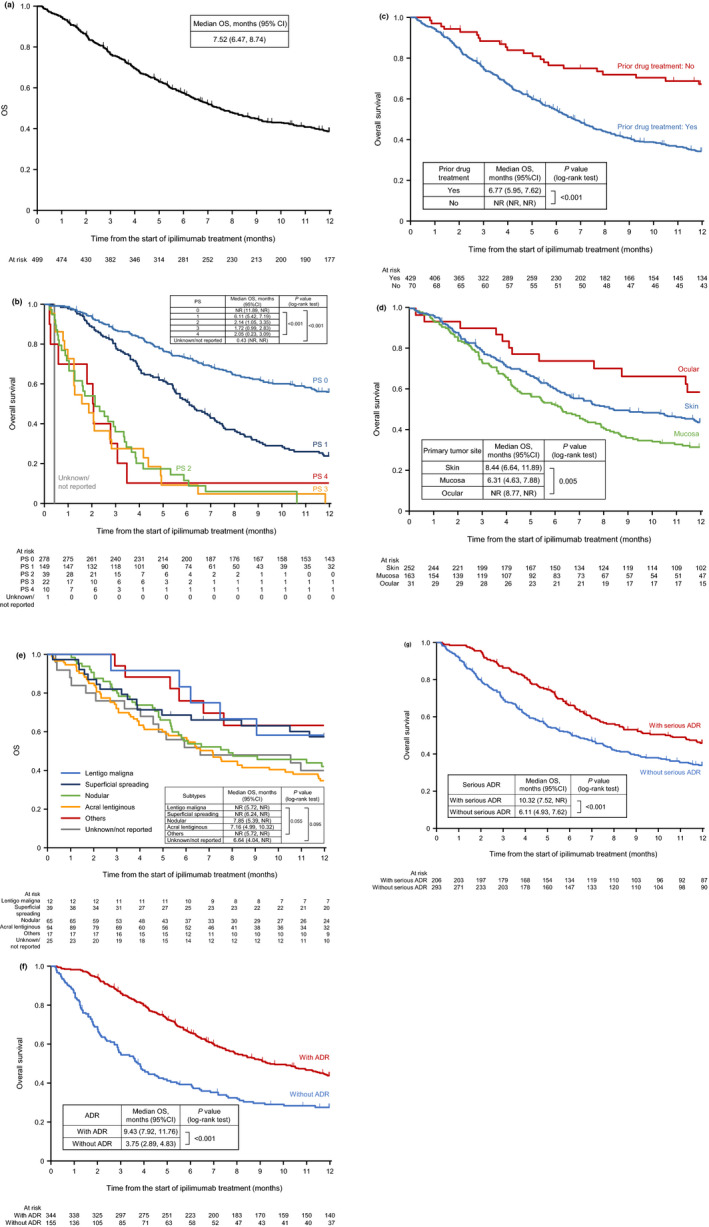
(a) Overall survival (Kaplan–Meier).^†^ (b) Overall survival by performance status. (c) Overall survival by prior drug treatment. (d) Overall survival by primary tumor site. (e) Overall survival by tumor subtypes of melanoma of the skin. (f) Overall survival by presence or absence of adverse drug reactions (ADR). (g) Overall survival by presence or absence of serious ADR. ^†^Observation of survival was censored at 12 months. CI, confidence interval; NR, not reached; OS, overall survival; PS, performance status.

The OS estimated by the Kaplan–Meier method is shown in Figure 5(a). The median OS was 7.52 months (95% CI, 6.4–8.74). Patients with a PS of 0 and 1 had longer OS than those with a PS of 2, 3 or 4 (Fig. 5b). Patients without prior drug treatment had longer OS than those who had received prior treatment (Fig. 5c). By primary tumor site, patients with tumors located in the mucosa had a shorter OS than those with skin or ocular tumors (Fig. 5d). The median OS was 7.85 months (95% CI, 5.39–not reached [NR]) in nodular melanoma, 7.16 months (95% CI, 4.99–10.32) in acral lentiginous melanoma, and NR in lentigo maligna melanoma (95% CI, 5.72–NR) and superficial spreading melanoma (95% CI, 6.24–NR) (Fig. 5e). There were no statistically significant differences among melanoma subtypes. OS was longer with the presence of ADR (Fig. 5f) and serious ADR (Fig. 5g).

## Discussion

As there were limited data on the safety and efficacy of ipilimumab in Japanese patients, the regulatory entity requested the conduct of this postmarketing surveillance of ipilimumab for Japanese patients with radically unresectable malignant melanoma in the real‐world clinical setting. In the studied population, most patients were more than 60 years of age, had an ECOG PS of 0 or 1, half had melanoma of the skin (mainly of the ALM subtype) and approximately three‐quarters had negative *BRAF* mutation status. Most patients had received multiple treatments as well as several rounds of pharmacotherapy with nivolumab, dacarbazine or vemurafenib, including third‐line ipilimumab in nearly half of the patients, and most had experienced recurrence. The characteristics of the Japanese population in this postmarketing surveillance are consistent with the previously reported characteristics in epidemiological studies of malignant melanoma and skin cancer in Japan.^2^, ^3^

Regarding the safety results, the overall incidence rate of ADR was 69.5%, and that of serious ADR was 40.8%. The most common ADR and serious ADR were liver disorder, colitis and diarrhea. The most common ADRI were liver‐related ADR (22.5%), skin‐related ADR (22.1%), gastrointestinal‐related ADR (20.3%) and endocrine system‐related ADR (16.3%). The present incidence rates seemed slightly higher than the rates observed in the Japanese phase II study in which the incidence of ADR was 60% (12/20 patients).^10^ Meanwhile, the present incidence rates were lower than the ADR incidence rate observed in the global phase III study in which the ADR incidence rate was 80.2% (105/131).^9^ As the Japanese phase II study included only 20 patients,^10^ we consider that the ADR incidences observed in the present postmarketing surveillance results are within the expected range for a study of this nature with a considerably larger sample size. Accordingly, no major changes were noted in the risk–benefit profile of ipilimumab and no new safety concerns were raised; thus, the safety profile of ipilimumab was further validated. Further, the ADR observed were mostly irAE; this was expected based on the mechanism of action of ipilimumab.

The most frequently reported ADRI occurred within 8 weeks, but endocrine system‐related ADR tended to occur at 8 weeks or later. Moreover, many of these endocrine system‐related ADR occurred after the fourth dose, while most of the other ADR were found to have occurred before the fourth dose when analyzing the incidence of ADR at each dose (Fig. 4). This relatively later occurrence of endocrine system‐related ADR may be a unique characteristic of ipilimumab compared with other programmed cell death 1/programmed death ligand 1 inhibitors, such as nivolumab.^13^ In the present study, the most common ADRI with more than 2%, which also tended to be serious ADR, were liver disorder, hepatic function abnormal, and aspartate aminotransferase and alanine aminotransferase increased; rash, pruritus and skin disorder; diarrhea, colitis and enterocolitis; and hypothyroidism, hypopituitarism, adrenal insufficiency and hypophysitis. Notably, the vast majority of these events had recovered or were in remission by the time of the last evaluation.

Regarding ipilimumab efficacy, among the 547 patients analyzed, 201 survived (36.7%) at 12 months after the start of the treatment. The median OS estimated with the Kaplan–Meier method was 7.52 months. Mucosal melanoma seems to be less sensitive to ipilimumab compared with melanoma of the skin. This trend differed from that observed in the nivolumab postmarketing surveillance,^14^ in which mucosal melanoma and melanoma of the skin had a similar sensitivity to treatment. These differences could be explained by differences in mutation patterns and these patients could perhaps benefit from nivolumab and ipilimumab combination therapy.^15^, ^16^

Overall, the OS observed in the present postmarketing surveillance (7.52 months) was similar to that reported in the Japanese phase II study evaluating ipilimumab monotherapy (median OS, 8.71 months)^10^ and other overseas real‐world data (median OS, 6.1–7.2 months);^17^, ^18^, ^19^ however, it was slightly shorter than that in the global phase III study (median OS, 10.1 months).^9^ The clinical studies enrolled patients with PS 0–1. In contrast, patients with PS 0–1 accounted for 85.7% of the patients in this postmarketing surveillance, while 14.1% of patients had a PS of 2 or higher.

Patients in later lines of treatment seem to be less sensitive to ipilimumab than first‐line or treatment‐naïve patients. A retrospective study analyzed OS in treatment‐naïve and previously treated patients who were treated with ipilimumab 10 mg/kg every 3 weeks for four doses. Notably, treatment‐naïve patients achieved better survival rates (69.4%, 62.9% and 56.9% at 12, 18 and 24 months, respectively) than previously treated patients (50.0%, 37.7% and 28.5% at 12, 18 and 24 months, respectively).^20^

Patients with ADR and serious ADR showed higher efficacy than patients without ADR. Based on recent reports on nivolumab, another immune checkpoint inhibitor, it has been suggested that the onset of immune‐related ADR of immune checkpoint inhibitors is associated with clinical benefit for these patients.^21^, ^22^, ^23^ Our findings support this hypothesis.

In this postmarketing surveillance, 44.6% of patients received four doses of ipilimumab, and patients received a median of three doses overall. The number of ipilimumab doses seems to be different in patients receiving ipilimumab as first‐ and later‐line treatment. In the present study, 66.7% of patients received four doses and had no prior treatments while the proportion of those with one and two or more previous treatments who received four doses of ipilimumab was 42.9% and 39.4%. The main reason for discontinuation was ADR (52.6%), followed by disease progression or onset of new lesion (32.8%), and death due to the primary disease (13.6%). Ipilimumab is used to treat patients at later stages of the disease, at which point, most patients will have received several lines of treatment; this could affect the efficacy of ipilimumab. In this postmarketing surveillance, 78.2% of patients had been treated with nivolumab before ipilimumab. Ipilimumab seems to be used mainly as second‐ or later‐line treatment in patients at later stages of the disease. The fact that nivolumab was approved in Japan earlier than ipilimumab may be one reason. Tsutsumida *et al.* ^24^ reported the actual situation of sequential treatment patterns of ipilimumab and nivolumab in Japan. They reported on 61 of 68 patients treated with nivolumab before ipilimumab, and seven of 68 patients treated with ipilimumab before nivolumab. Among patients who were switched from nivolumab to ipilimumab, the most common reason for switching treatment was progressive disease.

In a global phase III study (CheckMate 067),^25^ treatment‐naïve patients with advanced melanoma were randomly assigned to receive nivolumab plus ipilimumab, nivolumab alone or ipilimumab alone. At 5 years, the OS in the nivolumab plus ipilimumab group was 52%; in the nivolumab group, OS was 44%; and in the ipilimumab group, OS was 26%.^26^ These monotherapy groups showed improved results compared with previous studies, and this may have been caused by the treatment cross‐over observed among the groups. In a multicenter, single‐arm Japanese study, nivolumab plus ipilimumab also showed increasing survival of treatment‐naïve Japanese patients with unresectable stage III/IV or recurrent melanoma.^27^ Based on these studies, the combination of nivolumab and ipilimumab has been approved in Japan since 2018.^25^ In Japan, further studies are needed not only to establish the optimal treatment of advanced malignant melanoma in order to improve patient survival and prognosis but also to clarify the optimal treatment pattern of ipilimumab, nivolumab and ipilimumab plus nivolumab to yield the most benefits for these patients with fewer ADR.

### Limitations

This study was a non‐interventional observational study with no control group and data were collected by physicians in usual clinical practice. Therefore, physicians’ implementation of data collection might have introduced bias, and the types of safety and effectiveness data were limited compared with interventional clinical trials. Possible misclassifications of events cannot be ruled out because events were assessed by treating physicians and were not confirmed by an independent adjudication committee.

### Conclusions

Based on the incidence of ADR and serious ADR, ipilimumab was found to be tolerable and no new safety concerns were raised. Ipilimumab showed efficacy in improving the median OS of patients according to its condition of approval. Notably, patients receiving ipilimumab as later lines of treatment were less sensitive to ipilimumab. Additionally, those with mucosal melanoma seemed to be less sensitive than those with skin type melanoma, but relatively higher efficacy was observed in patients with ADR and serious ADR.

## Conflict of Interest

N. Y., Y. K., H. U. and T. T are members of the Nivolumab/Ipilimumab Appropriate Use Committee for Melanoma, which is sponsored by Ono Pharmaceuticals and Bristol‐Myers Squibb. N. Y. received research funding, speaker’s fees, conference registration fees and/or travel or accommodation expenses from Bristol‐Myers Squibb, Ono Pharmaceuticals, MSD and Novartis Pharma. Y. K. received research funding from Ono Pharmaceuticals and Bristol‐Myers Squibb, and speaker’s fees, conference registration fees and/or travel or accommodation expenses from Ono Pharmaceuticals, Bristol‐Myers Squibb and Chugai Pharmaceutical. H. U. received research funding from MSD, Ono Pharmaceuticals, Bristol‐Myers Squibb, Chugai Pharmaceutical, Novartis Pharma, Takara Bio and Kyowa Hakko Kirin; consultancy or commission fees from MSD, Ono Pharmaceuticals, Bristol‐Myers Squibb, Chugai Pharmaceutical, Novartis Pharma and Roche Diagnostics; a fellowship and/or research or education grants from Ono Pharmaceuticals and Mochida Pharmaceutical; and speaker’s fees from MSD, Ono Pharmaceuticals, Bristol‐Myers Squibb, Chugai Pharmaceutical and Novartis Pharma. T. T. received research funding from Ono Pharmaceuticals and Taiho Pharmaceutical, and consultancy or commission fees from Ono Pharmaceuticals and Bristol‐Myers Squibb. K. M., N. S., E. I. and A. K. are employees of Bristol‐Myers Squibb.
